# Human Serum Enhances Biomimicry of Engineered Tissue Models of Bone and Cancer

**DOI:** 10.3389/fbioe.2021.658472

**Published:** 2021-07-13

**Authors:** Aranzazu Villasante, Samuel T. Robinson, Andrew R. Cohen, Roberta Lock, X. Edward Guo, Gordana Vunjak-Novakovic

**Affiliations:** ^1^Department of Biomedical Engineering, Columbia University, New York, NY, United States; ^2^Institute for Bioengineering of Catalonia (IBEC), The Barcelona Institute of Science and Technology (BIST), Barcelona, Spain; ^3^Department of Electronics and Biomedical Engineering, University of Barcelona, Barcelona, Spain; ^4^Bone Bioengineering Laboratory, Department of Biomedical Engineering, Columbia University, New York, NY, United States; ^5^Department of Electrical and Computer Engineering, College of Engineering, Drexel University, Philadelphia, PA, United States; ^6^Department of Medicine, Columbia University, New York, NY, United States; ^7^College of Dental Medicine, Columbia University, New York, NY, United States

**Keywords:** Ewing’s sarcoma, 3D cancer models, 3Rs, human serum, cypridina luciferase

## Abstract

For decades, fetal bovine serum (FBS) has been used routinely for culturing many cell types, based on its empirically demonstrated effects on cell growth, and the lack of suitable non-xenogeneic alternatives. The FBS-based culture media do not represent the human physiological conditions, and can compromise biomimicry of preclinical models. To recapitulate *in vitro* the features of human bone and bone cancer, we investigated the effects of human serum and human platelet lysate on modeling osteogenesis, osteoclastogenesis, and bone cancer in two-dimensional (2D) and three-dimensional (3D) settings. For monitoring tumor growth within tissue-engineered bone in a non-destructive fashion, we generated cancer cell lines expressing and secreting luciferase. Culture media containing human serum enhanced osteogenesis and osteoclasts differentiation, and provided a more realistic *in vitro* mimic of human cancer cell proliferation. When human serum was used for building 3D engineered bone, the tissue recapitulated bone homeostasis and response to bisphosphonates observed in native bone. We found disparities in cell behavior and drug responses between the metastatic and primary cancer cells cultured in the bone niche, with the effectiveness of bisphosphonates observed only in metastatic models. Overall, these data support the utility of human serum for bioengineering of bone and bone cancers.

## Introduction

Fetal bovine serum (FBS) is the gold standard for cell culture because of its documented effects on cell growth (van der Valk et al., 2018). However, the use of animal serum in cell culture medium can have a number of disadvantages (Gstraunthaler et al., 2013; van der Valk et al., 2018), including safety concerns associated with bovine diseases such as viral contaminants, prion proteins or mycoplasma, and batch-to-batch variations in FBS composition. Also, the use of animal serum does not necessarily represent the human condition, limiting the predictability of experimental outcomes (Gstraunthaler et al., 2013; van der Valk et al., 2018). In addition, there are concerns regarding animal welfare in terms of the 3R principle (Replacement, Reduction, and Refinement), as FBS is obtained from calf fetuses by cardiac puncture from pregnant cows during slaughter, and implies the sacrifice of large number of animals with fetal distress (Gstraunthaler et al., 2013; van der Valk et al., 2018). To overcome these issues, human serum and human platelet lysate have been suggested as FBS alternatives to enhance safety, quality and reproducibility of *in vitro* assays, and reduce animal suffering.

It is reported that cell responses can be different depending on the exposure to human serum, FBS, or human platelet lysate. Specifically, human serum has been shown to modify cancer cell behavior by enhancing cell invasion and spheroid formation in cervical cancer, in comparison to FBS (Heger et al., 2018). The use of human serum supported the growth and osteogenic differentiation of primary cells, such as bone marrow mesenchymal stem cells (Shahdadfar et al., 2005), human periosteum-derived cells (Roberts et al., 2014), human dental pulp stem cells (Pisciotta et al., 2012), and human adipose-derived stem cells (de Paula et al., 2013), and maintained the growth of human endothelial cells (Canovas and Bird, 2012).

Human platelet lysate (hP) is an alternative to FBS for culturing mesenchymal stem cells (Hemeda et al., 2014; Shanbhag et al., 2017; Ren et al., 2018; Saury et al., 2018), monocytes (Barsotti et al., 2013; Svajger, 2017), human umbilical vein endothelial cells (HUVECs) (Barsotti et al., 2013; Hofbauer et al., 2014; Robinson et al., 2016), and cell lines for leukemia, colon cancer (Pons et al., 2018) and hepatocellular carcinoma (Carr et al., 2014). Interestingly, the use of hP has been also reported for tissue engineering. For example, hP enabled osteogenic differentiation of different types of mesenchymal stem cells for bone regeneration (Shanbhag et al., 2017), and supported generation of dendritic cells from monocytes (Svajger, 2017).

However, in spite of evidence that human derivatives can provide promising alternatives to FBS for mimicking human tumor microenvironments, research is still mostly done using animal supplements. Therefore, the published data for the use of human derivatives in studies of cancer and *in vitro* 3D tumor modeling are limited.

We hypothesized that human serum will improve biomimicry of human bone for studies of tissue-engineered bone tumors. To this end, we analyzed the effects of human serum and human platelet lysate on the functional capacity of human MSC-derived osteoblasts and monocytes-derived osteoclasts. We also investigated engineering of human tumors that normally reside in bone, using Ewing’s sarcoma (EWS) as a representative model.

Ewing’s sarcoma is a poorly differentiated pediatric tumor of aggressive behavior characterized by a propensity to metastasize to bone (Lawlor and Sorensen, 2015; Redini and Heymann, 2015). In the bone niche, EWS grows fast and induces progressive bone destruction by osteolysis. However, EWS cells are incapable to directly degrade bone matrix. Instead, they orchestrate the process of bone resorption through a vicious cycle of recruitment and activation of osteoclasts that is mediated by osteoblasts (Redini and Heymann, 2015). Bone destruction releases calcium and growth factors from the bone matrix that favor acidosis and osteoclasts differentiation and activation that in turn cause osteolytic lesions, broken bones, pain and hypercalcemia (Redini and Heymann, 2015).

We evaluated cell growth and osteolysis of the metastatic and primary EWS cells in the presence of human supplements. For determining cancer cell growth, we implemented a non-destructive strategy based on secretion of luciferase from labeled cells. We also studied cancer cell responses to two bisphosphonates: Zoledronic acid (ZA) and Alendronate (ALD) that have been shown effective in inhibiting osteolysis in humans and preclinical studies with animals, as well as affecting EWS cell proliferation (Sonnemann et al., 2003; Zhou et al., 2005; Odri et al., 2014; Redini and Heymann, 2015; Heymann et al., 2020).

## Materials and Methods

### Cell Culture

#### Cancer Cell Lines

Bone metastatic Ewing’s sarcoma cell line SK-N-MC (HTB-10) and primary Ewing’s sarcoma cell line RD-ES (HTB-166) were purchased from the American Type Culture Collection (ATCC) and cultured according to the manufacturer’s specifications. RD-ES cells were cultured in ATCC-formulated RPMI-1640 Medium (RPMI) and SK-N-MC cells were cultured in ATCC-formulated Eagle’s Minimum Essential Medium (EMEM). Both media were supplemented with 1% penicillin/streptomycin, and either 10% (v/v) Hyclone FBS, 10% (v/v) human serum (Corning, 35-060), or 5% (v/v) Human Platelet Lysate (Stemcell, 6961). Cells were cultured at 37°C in a humidified incubator at 5% CO_2_.

#### Generation of Cypridina Luciferase-Expressing Cell Lines

Generation of Ewing’s sarcoma cells lines expressing cypridina luciferase (cLuc) were performed by lentiviral transduction using cLuc-RFP-Puro lentiviral particles (GenTarget Inc., LVP375, 1 × 107 IFU/ml). Cancer cells (50,000 per well) were cultured in a 24 well plates overnight in EMEM or RPMI media supplemented with 1% penicillin/streptomycin and 10% (v/v) Hyclone FBS. Culture medium was then removed and 1 mL of fresh medium containing 50 μL of cLuc-RFP-Puro lentiviral particles was added to cells. After 48 h of incubation, cells were selected for 3 days with 2 μg/mL of puromycin (Sigma, P8833) to generate a stable cell line.

#### Tumor Aggregates

To generate tumor aggregates, aliquots of 300,000 cLuc- Ewing’s sarcoma cells were centrifuged in 15 ml Falcon tubes (5 min at 12,000 rpm), and cultured in 4 mL of Minimum Essential Medium Eagle Alpha modification (α-MEM, Sigma, M4526) supplemented with 10% (v/v) human serum, 1% penicillin/streptomycin and L-Glutamine (Gibco #25030-081) for 1 week.

#### Human Mesenchymal Stem Cells and Osteoblasts Differentiation

Human mesenchymal stem cells (hMSC) were purchased from Lonza and characterized and prepared as in our previous studies (Villasante et al., 2014, 2017). For hMSC expansion, cells were cultured in basic medium (DMEM supplemented with 10% (v/v) Hyclone FBS, 1 ng/mL of basic fibroblast growth factor b, bFGF and 1% penicillin/streptomycin). For studies of differentiation into osteoblasts, hMSC were cultured in a differentiation medium that consists in DMEM supplemented with either 10% (v/v) Hyclone FBS, 10% (v/v) human serum (Corning, 35-060), or 5% (v/v) Human Platelet Lysate (Stemcell, 6961), and 1 μM dexamethasone, 10 mM β-glycerophosphate, and 50 μM ascorbic acid-2-phosphate. All cells were cultured at 37°C in a humidified incubator at 5% CO_2_.

#### Isolation of Monocytes and Osteoclast Differentiation

Peripheral blood mononuclear cells (PBMC) were isolated from buffy coats of human blood obtained from the New York Blood Center, by density gradient centrifugation with Ficoll-paque PLUS (GE Healthcare, #17-1440-02). CD14^+^ monocytes were then isolated from the PBMC preparations by using Percoll (GE Healthcare, #17-0891-02) density gradient centrifugations, as we have previously described (Villasante et al., 2017). Then, 8 × 10^6^ CD14^+^ monocytes were cultured on 25 cm^2^ ultra-low attachment flasks (Corning #3815) with 10 mL of maintenance medium: RPMI 1640 (ATCC, 30-2001) supplemented with 10% heat inactivated human serum (Corning #35-060), 1% penicillin/streptomycin, 20 ng/ml Recombinant Human M-CSF (Prepotech #300-25) during 2 days at 37°C in a humidified incubator at 5% CO_2_.

For derivation of osteoclasts from human monocytes, human CD14^+^ monocytes were incubated with differentiation medium consisting of Minimum Essential Medium Eagle Alpha modification (α-MEM, Sigma, M4526) supplemented with either 10% (v/v) heat inactivated Hyclone FBS, 10% (v/v) heat inactivated human serum (Corning, 35-060), or 5% (v/v) human Platelet Lysate (Stemcell, 6961), 1% penicillin/streptomycin, L-Glutamine (Gibco #25030-081), 20 ng/ml Recombinant Human M-CSF (Prepotech #300-25) and 40 ng/ml Recombinant Human sRANK Ligand (Prepotech #310-01). Cytokines were replenished every 3 days. Cells were maintained at 37°C in a humidified incubator at 5% CO_2_.

### Engineering Bone Tissue

Bone scaffolds were prepared from decellularized bovine bone as in our previous studies (Villasante et al., 2014, 2017). Briefly, bovine wrists (Green Village Packing) were cut into axial sections using a vertical bandsaw, and machined with a standard 2 flute endmill to a final geometry of 4 mm × 4 mm × 1 mm (length × depth × thickness). Decellularization of bone was performed by sequential detergent washes [EDTA (0.1%) in PBS, EDTA (0.1%) in Tris (10 mM), and SDS (0.5%) in Tris (10 mM)], followed by treatment by DNase and RNase in Tris buffer (10 mM) to completely remove remaining biological components. hMSC (0.75 × 10^6^ per scaffold) were seeded into each scaffold and cultured with osteoblast differentiation medium for 3 weeks, with medium change twice a week.

Bone constructs were incubated in osteoclast differentiation medium without cytokines (M-CSF and sRANK Ligand) for 1 h and then cultured with the addition of 0.25 × 10^6^ CD14^+^ monocytes in 10 μl of osteoclast differentiation medium for 30 min at 37°C in a humidified incubator at 5% CO_2_. The constructs were flipped and seeded again with 0.25 × 10^6^ CD14^+^ monocytes in 10 μl of osteoclast differentiation medium for 30 min at 37°C in a humidified incubator at 5% CO_2_. The resulting constructs were placed into low attachment twenty-four well plates (1 construct per well) containing 1 ml of osteoclast differentiation medium. Medium was changed twice a week.

### Tissue-Engineered Tumor Models

Tumor cells were introduced into the bone niche using methods from our previous studies (Villasante et al., 2014). Aggregates of Ewing’s sarcoma cells (RD-ES or SK-N-MC cell line) containing 0.3 × 10^6^ cells were injected into the tissue constructs (1 aggregate per construct) and the resulting cancer cell-bone constructs were cultured for 1 week in osteoclast differentiation medium without supplemental cytokines.

### Cancer Cell Proliferation Assays

#### MTS Assay

MTS cell proliferation assay in monolayer was performed using CellTiter 96^®^ AQueous One Solution kit (Promega, G3582), following the manufacturer’s protocol.

#### Cypridina Luciferase Assay

Real-time and time-course cell proliferation, for cancer cells in monolayer and within the 3-dimensional TE-bone, were performed using the Pierce^TM^ Cypridina Luciferase Glow Assay Kit (ThermoFisher, #16170), according to the manufacture’s protocol. Briefly, working solution was prepared by adding 10 μL of 100X vargulin to 1 mL of Cypridina Glow Assay Buffer. Then, 10 μL of each diluted cell supernatant were added to a white opaque 96-well plate (Corning, #3912) and right after, 50 μL of working solution was added to each well for detection of cLuc activity by using a luminometer.

### Resorption Pit Assay

Human CD14^+^ monocytes were plated into 24-well osteo assay plate (100,000 cells per well) (Corning, #3987) and cultured either in complete osteoclast differentiation medium, or without sRANKL as a control for cell differentiation. At different time points, 10% bleach solution was added to each well and cells were incubated for 10 min at room temperature. Then, wells were washed 3 times with distilled water and air dried over night. Resorption pits were visualized at 10× magnification and, for improving the quality of the image, a blue filter was used.

### Quantitative Real-Time PCR

Quantitative real time-PCR (qRT-PCR) was carried out using DNA Master SYBR Green I mix (Applied Biosystems), as we previously described (Villasante et al., 2014) mRNA expression levels were quantified applying the ΔCt method, ΔCt = (Ct of gene of interest—Ct of GAPDH). qRT-PCR primer sequences that were obtained from the PrimerBank data base^1^ are listed in Table 1. Primer sequences for CD206, CD163, CCR7, CD80, and TNFα were obtained from Spiller et al. (2014).

**TABLE 1:** PrimerBank IDs of primer sequences used for qRT-PCR.

Gene	PrimerBank ID
Cathepsin K	315075295c1
TRAP	161377452c1
CD14	291575162c1
COL1A1	110349771b2
BSP	167466186b1
OPN	38146097b1
BGLAP	158517828b1

### Histology and Immunohistochemistry

Tumor tissue constructs and all controls were washed in PBS, fixed in 10% formalin for 24 h. The samples were processed for histological and immunohistochemical analysis by decalcifying with Immunocal (StatLab Corp., McKinney, TX, Unite States) for 2 days, and right after they were dehydrated in graded ethanol washes, and embedded in paraffin. Serial sections (5 μm thick) were mounted on glass slides and stained by using routine hematoxylin/eosin and Masson trichrome procedures.

#### Immunohistochemistries

Immunohistochemistries were performed using primary antibodies specific to CD99 (dilution 1:500; Signet antibodies, SIG-3620) and bone sialoprotein (dilution 1:500, Abcam, ab33022), and developed using the Vector Elite ABC kit (Vector Laboratories), following manufacturer instructions. Briefly, sections were blocked with serum for 30 min and incubated with the primary antibody overnight at 4°C. After washing with PBS, samples were incubated with secondary antibodies and developed (Vector Laboratories). Negative controls were prepared by omitting the primary antibody step.

#### Alkaline Phosphatase and von Kossa Stainings

Alkaline phosphatase and von Kossa stainings were performed as previously described (Falcone and Casucci, 2016).

#### Tartrate-Resistant Acidic Phosphatase Staining

Tartrate-Resistant Acidic Phosphatase staining was performed using the K-assay (Kamiya Biomedical Company #KY-008).

For staining osteoclasts in monolayer, 300,000 monocytes per well in 6-well plates were cultured in complete osteoclast differentiation medium (with FBS, human serum or human Platelet Lysate), or without sRANKL as a control for differentiation. At time intervals (1, 2, and 3 weeks), culture medium was removed and cells were fixed and stained for TRAP, by following the manufacturer’s protocol.

For TRAP staining in 3D, tissue-engineered bone constructs were fixed in 10% formalin, decalcified in 12.5% EDTA, embedded in paraffin, sectioned to 4 μm, stained for TRAP according to the manufacturer’s instructions, and counterstained with Hematoxylin QS (Vector Labs).

### Calcium Release Analysis

Supernatants of culture medium were sampled (1 mL per sample), snap frozen in liquid nitrogen and stored at −80 °C. The Ca2+ concentrations were analyzed using the Ca2+ Detection Kit (Abcam, ab102505) following the manufacturer protocol. Briefly, supernatants were centrifuged for 2–5 min at 4°C at top speed using a cold microcentrifuge to remove any insoluble material. Supernatants were collected and transferred to clean tubes. 90 μL of the chromogenic reagent were added to each sample. The chromogenic complex formed between calcium ions and 0-cresolphthalein was measured using a microplate reader at OD = 575 nm. The measured absorbance values for each standard were plotted as a function of the final concentration of calcium, and the calcium concentrations in the samples were calculated from the standard curve.

### Osteopontin and Osteocalcin Release Analyses

Supernatants from untreated and treated (with zoledronic acid or alendronate) TE-ES were analyzed to detect osteopontin and osteocalcin proteins, using a human osteopontin (OPN) Quantikine ELISA Kit (R&D systems, DOST00), and a human Osteocalcin Quantikine ELISA Kit (R&D systems, DSTCN0) according to the manufacturer’s instructions.

### Micro-Computed Tomography

Samples were scanned using a Scanco vivaCT 80 system (Scanco Medical AG, Brüttisellen, Switzerland) using energy parameters of 55 kVp, 145 μA, 1,000 projections and an integration time of 200 ms. Scans were reconstructed at an isotropic voxel size of 21 μm. Grayscale images were smoothed using a Gaussian filter (σ = 0.8, support = 1), and segmented using a threshold of 32% of the maximum grayscale value. All image processing and analyses were done using standard Scanco software.

## Results

### Evaluation of Human Serum and Human Platelet Lysate as Substitutes of FBS for Osteoblasts and Osteoclasts Differentiation

**FIGURE 1:**
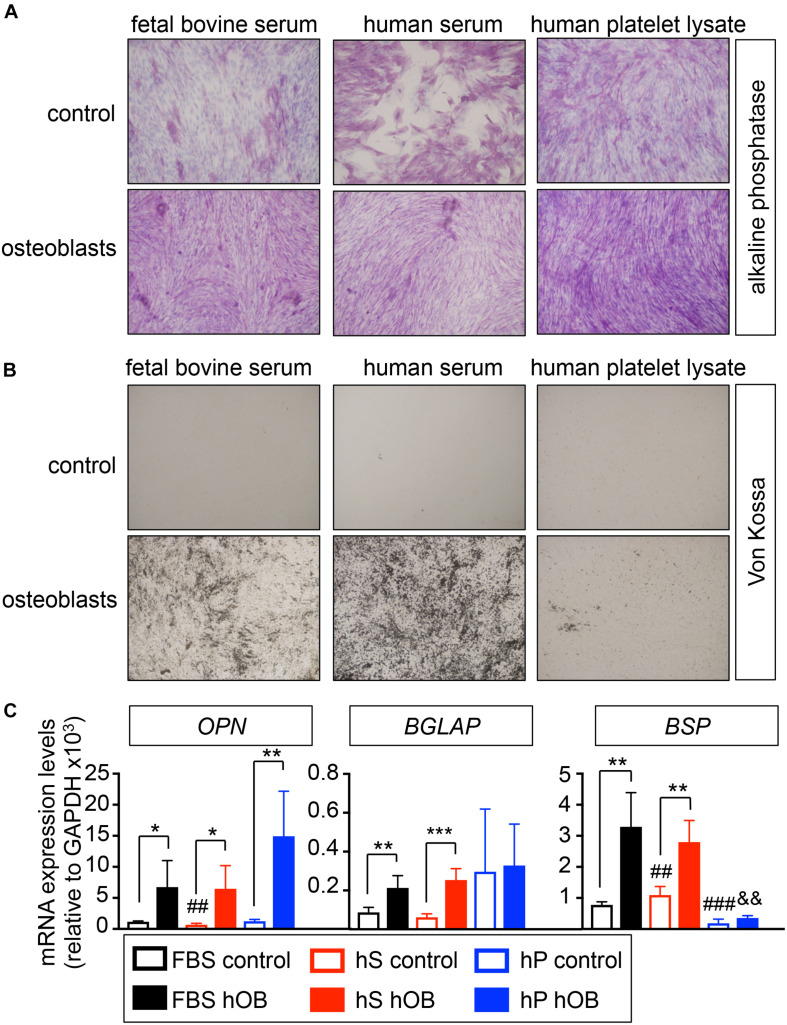
Evaluation of the capability of human derivatives to support differentiation of human mesenchymal stem cells into osteoblasts in monolayer. **(A)** Early osteogenic differentiation was confirmed by Alkaline phosphatase staining (ALP). Human mesenchymal stem cells were cultured in osteoblasts differentiation medium, or control medium, supplemented with FBS, human serum or human platelet lysate for 3 weeks before stain. Representative images of cell staining for each group are shown (*n* = 3 per group and donor. hMSC donors = 2). **(B)** Von Kossa staining for demonstrating deposits of calcium in each group. The cells positively stained for Von Kossa are shown in black/brown (*n* = 3 per group and donor. hMSC donors = 2). **(C)** qRT-PCR analysis of osteoblasts differentiation genes OPN, BGLAP and BSP. FBS control; basic medium-containing FBS (*n* = 4); hS control, basic medium-containing human serum (*n* = 4); hP control, basic medium-containing human platelet lysate (*n* = 4). FBS hOB, differentiation medium-containing FBS (*n* = 4); hS hOB, differentiation medium-containing human serum (*n* = 4); hP hOB, differentiation medium-containing human platelet lysate (*n* = 4). Fold change was calculated by normalizing to GAPDH levels. Values correspond to the average and standard deviation. Two-tailed Student’s *t*-test was used to determine statistical significance. *p < 0.05, **p < 0.01, and ***p < 0.001 for each hOB medium compared to its respective medium control; ^#^^#^*p* < 0.01 for hS control compared to FBS control; ^#^^#^^#^*p* < 0.001 for hp control compared to FBS control; ^&^^&^*p* < 0.01 for hS hOB compared to FBS hOB.

We first studied the capability of human mesenchymal stem cells (hMSC) to differentiate into osteoblasts in osteogenic differentiation medium supplemented with human serum (hS-OB) or human platelet lysate (hP-OB), in comparison to FBS-containing medium (FBS-OB). Osteoblasts differentiation in 2D was evaluated by alkaline phosphatase staining, von Kossa staining and osteoblasts-related gene expression (Figure 1). We observed alkaline phosphatase positive cells in hS-OB, hP-OB, and FBS-OB cultures (Figure 1A). Unexpectedly, control medium with hS induced alkaline phosphatase in the whole cell culture (Figure 1A). We only observed homogeneous von Kossa positive cell cultures in the hS-OB and FBS-OB conditions (Figure 1B). This result eliminates hP as an alternative to FBS for osteoblasts differentiation. hS-OB also supported the expression of the bone-related genes *OPN, BGLAP* and *BSP* at similar levels to FBS-OB (Figure 1B). Although hP-OB enhanced *OPN* expression, it did not have an effect on *BGLAP* or *BSP*, which reinforces the fact that hP is not an effective substitute of FBS for osteoblast differentiation. This study indicates that hS could be used to support hMSC differentiation into osteoblasts, as an alternative to FBS.

**FIGURE 2:**
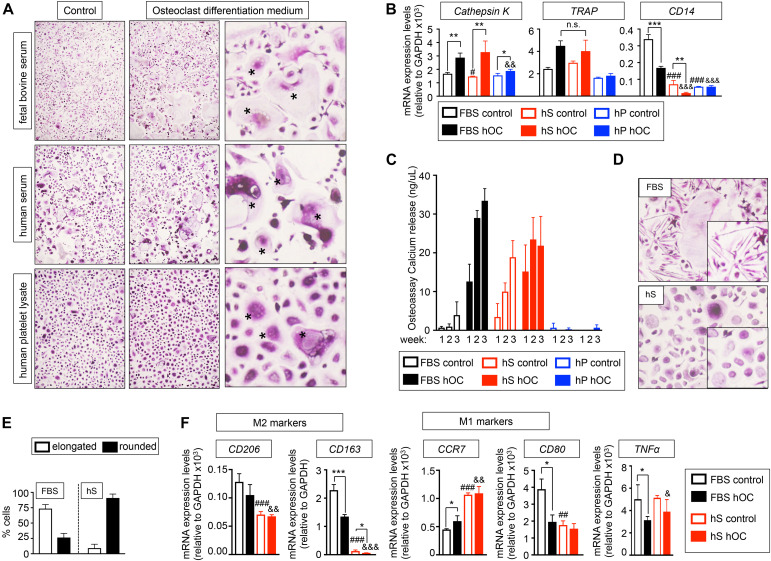
Evaluation of the capability of human derivatives to support Differentiation of human monocytes CD14^+^ into osteoclasts in monolayer. **(A)** TRAP staining of monocytes CD14^+^ cultured in maintenance medium (control) or osteoclasts differentiation medium (+RANKL) for 3 weeks. Both control and differentiation media were supplemented with the indicated serum derivatives. Right panel, asterisks show the typical morphology of osteoclasts, after seeding and differentiation of osteoclasts precursors. Representative images of TRAP staining for each group are shown (*n* = 3 per group and donor. monocytes CD14^+^ donors = 3). **(B)** Analysis of mRNA levels of osteoclasts markers by qRT-PCR at week 3 of monocytes CD14^+^ maturation. Comparison of six different culture media: FBS, Fetal bovine serum; hS, human serum; hP, human platelet lysate; control, monocytes CD14^+^ maintenance medium; hOC, osteoclasts differentiation medium (+RANKL); *n* = 3 per condition. Relative endogenous expression of osteoclasts markers was normalized to GAPDH levels. Error bars represent standard deviation of relative expression. Two-tailed Student’s *t*-test was used to determine statistical significance. *p < 0.05, **p < 0.01 and ***p < 0.001 for each hOC medium compared to its respective medium control; ^#^*p* < 0.05 for hS control compared to FBS control; ^#^^#^^#^*p* < 0.001 for hS or hp control compared to FBS control; ^&^^&^*p* < 0.01 for hP hOC compared to FBS hOC; ^&^^&^^&^*p* < 0.001 for hS or hp hOC compared to FBS hOC; n.s., not significant. **(C)** Calcium release levels from monocytes CD14^+^ cultured on osteoassay plates at indicated culture media and time points. **(D)** Representative TRAP staining detail of monocytes CD14^+^ cultured in osteoclasts differentiation medium containing FBS or hS showing differences in cell morphology (elongated in FBS-medium and rounded in hS-medium). **(E)** Quantification of the percentage of elongated or rounded cells present in osteoclasts differentiation medium containing FBS or hS. **(F)** Expression levels of M2 and M1 macrophages markers by qRT-PCR in both control and osteoclast differentiation (hOC) medium supplemented with FBS or human serum (hS) at week 3 of monocytes CD14+ differentiation. mRNA levels of M1 and M2 markers were normalized to GAPDH levels. Error bars represent standard deviation of relative expression. Two-tailed Student’s *t*-test was used to determine statistical significance. *p < 0.05, **p < 0.01 for each hOC medium compared to its respective medium control; ^##^*p* < 0.01, ^###^*p* < 0.001 for hS control compared to FBS control; ^&^*p* < 0.05, ^&⁣&^*p* < 0.01, ^&⁣&⁣&^*p* < 0.001 for hS hOC compared to FBS hOC; ^&⁣&⁣&^*p* < 0.001 for hS hOC compared to FBS hOC.

To investigate if hS and hP enabled osteoclasts differentiation in 2D, we compared the effectiveness of human osteoclast differentiation medium (OC) supplemented with hS (hS-OC), hP (hP-OC) and FBS (FBS-OC) to differentiate monocytes from patients into mature osteoclasts, using our well-established protocol (Villasante et al., 2017). TRAP staining demonstrated the presence of large multinucleated TRAP-positive cells in cultures treated with FBS-OC and with hS-OC, but not with hP-OC (Figure 2A). hS-OC also induced expression of the TRAP gene and the Cathepsin K gene, which are both osteoclasts-related genes; no differences in TRAP expression between FBS-OC and hS-OC conditions were observed (Figure 2B). We found Cathepsin K expression in hP-OC cultured cells, as well, but at lower levels compared to FBS-OC and hS-OC (Figure 2B).

Next, we studied the expression of CD14, which is highly expressed in osteoclasts precursors (CD14^+^ monocytes) and is downregulated during osteoclast differentiation. Therefore, CD14 mRNA expression could be used as an indicator of the efficiency of osteoclast differentiation. We observed that culture medium supplemented with hS or hP downregulated CD14 to a lower level than the one observed for control-FBS and, surprisingly, FBS-OC. Importantly, hS-OC had a dramatic effect on suppressing CD14 expression (Figure 2B). These results suggest that osteoclasts precursors differentiate into osteoclasts more efficiently in the presence of hS than FBS.

To further evaluate osteoclasts differentiation and functionality, we differentiated CD14^+^ monocytes on osteo-assay plates. Osteoclasts generated with hS-OC and cultured on osteoassay plates released calcium, an indicator of maturity and function (Figure 2C). CD14^+^ monocytes cultured in control medium containing hS were also functional and released calcium at the same levels observed for hS-OC osteoclasts. We did not observe functional cells when hP was added to culture medium (Figure 2C). Therefore, our data confirm hS as a human alternative to FBS for osteoblast and osteoclast differentiation.

Human monocytes can polarize into a spectrum of phenotypes, such as M1 macrophages and M2 macrophages that play a role in inflammation, resistance to infection and tissue repair (Huang et al., 2017). M1 macrophages can also be found in healthy bones, a transient state similar to the transition of pre-osteoclasts into mature osteoclasts (Huang et al., 2017). Polarization of monocytes toward an M1 macrophage phenotype is associated with round cell shape, while M2 phenotype is associated with an elongated cell shape (McWhorter et al., 2013). We observed that FBS-OC medium favored elongated cells, while hS-OC promoted the round cell phenotype (Figures 2D,E). We hypothesized that FBS-OC could drive CD14^+^ monocytes toward the M2 macrophage phenotype, while the hS-OC medium will support the osteoclast precursors, which are of the M1 phenotype. To test this hypothesis, we analyzed the expression of M1 and M2-related genes, and confirmed that M2 genes are expressed at higher levels in FBS-OC treated CD14^+^ monocytes compared to hS-OC cells, with the opposite result for M1 markers (Figure 2F).

We concluded that hS can serve as an FBS substitute for osteogenic differentiation, and enable osteoclast differentiation more efficiency than FBS, presumably due to the induction of M1 macrophage phenotype and the *in vitro* generation of pre-osteoclasts. Thus, we chose human serum as the FBS alternative for bone tissue engineering.

### Generation and Validation of a Bone-Engineered Platform

**FIGURE 3:**
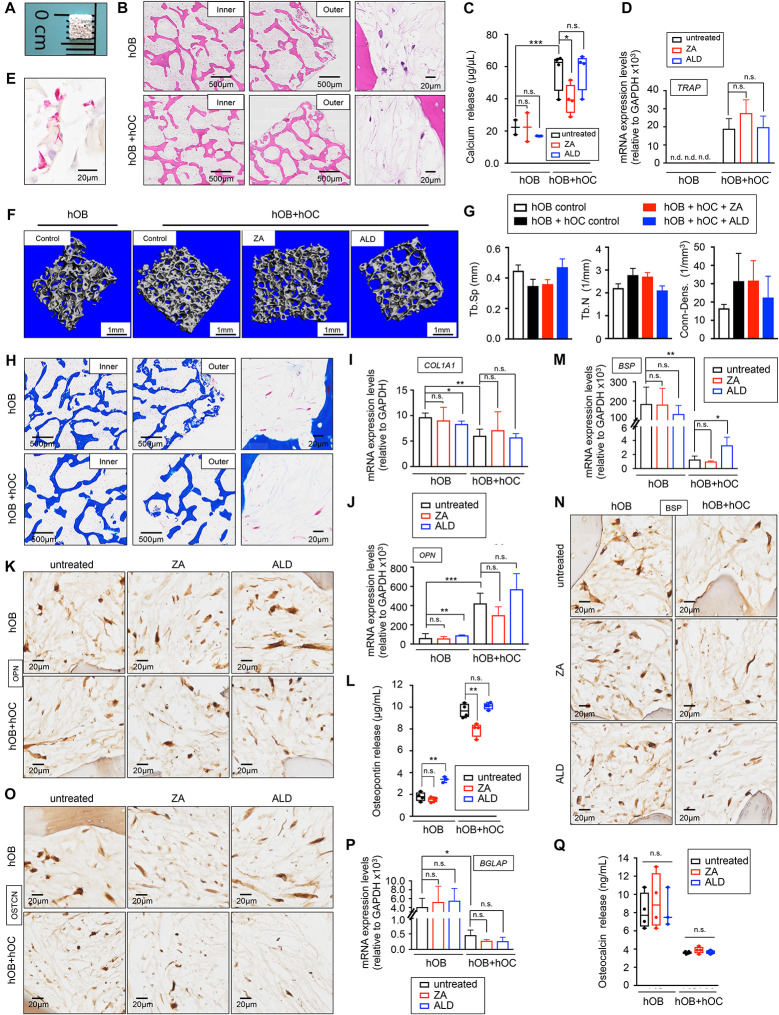
Healthy bone-engineered niche cultured in human serum-containing medium **(A)** Representative image of the decellularized bone scaffold 4 mm × 4 mm × 1 mm (length × depth × thickness). **(B)** Hematoxylin and Eosin stains of the inner and outer regions of the indicated tissue-engineered bones (TE-bones). Right panels show a detail of bone morphology of both models. hOB, tissue-engineered bone containing only human osteoblasts; hOB+hOC tissue-engineered bone containing human osteoblasts and human osteoclasts in co-culture. **(C)** Calcium release levels from the indicated TE-bones untreated (control) and treated with 20 μM Zoledronic acid (ZA) or 10 μM Alendronate (ALD) for 3 days. Two-tailed Student’s *t*-test was used to determine statistical significance. *p < 0.05; **p < 0.01, ***p < 0.001; *n* = 3–4; n.s., not significant. **(D)** Analysis of TRAP mRNA levels by qRT-PCR at week 2 of monocytes CD14+ maturation. Comparison of TE-bone made with osteoblasts only (hOB) and TE-bone containing both osteoblasts and osteoclasts (hOB+hOC) in untreated and ZA or ALD treated samples. Relative endogenous expression of osteoclasts markers was normalized to GAPDH levels. Error bars represent standard deviation of relative expression. Two-tailed Student’s *t*-test was used to determine statistical significance. n.d., not determined. n.s., not significant. hOB *n* = 4 for every condition. hOB + hOC *n* = 3 for every condition. **(E)** TRAP staining of osteoclasts in co-culture with osteoblasts within the TE-bone model hOB+hOC at week 2 of monocytes CD14^+^ differentiation. **(F)** Representative three-dimensional bone architecture from TE-bone constructs made with osteoblasts only (hOB) or both cell types (hOB+hOC) with or without bisphosphonates treatment obtained by micro CT imaging (*n* = 4 per group). **(G)** Plots of the structural parameters trabecular space (Tb.Sp), trabecular number (Tb.N) and connectivity density (Conn.Dens) from microCT images for the four groups examined (*n* = 4 per group). **(H)** Representative Masson’s trichrome staining images showing collagen in blue in the indicated regions of both TE-bone models. **(I)** qRT-PCR analysis of *COL1A1*. Fold change was calculated by normalizing to GAPDH levels in individual samples. Values correspond to the average and standard deviation. Two-tailed Student’s *t*-test was used to determine statistical significance. *p < 0.05; **p < 0.01; n.s., not significant. hOB for every indicated condition *n* = 4. hOB + hOC *n* = 3 for every indicated condition. **(J)** Expression levels of *OPN* by qRT-PCR in both TE-bone models and indicated treatments. mRNA levels were normalized to GAPDH levels. Error bars represent standard deviation of relative expression. Two-tailed Student’s *t*-test was used to determine statistical significance. **p < 0.01, ***p < 0.001; n.s., not significant. **(K)** Immunohistochemical staining of OPN at indicated TE models. Counterstaining was performed with hematoxylin QS (blue). **(L)** Osteopontin release levels from the TE-bone hOB and hOB+hOC at indicated conditions. Error bars represent standard deviation; *n* = 4 per condition. Two-tailed Student’s *t*-test was used to determine statistical significance. **p < 0.01; n.s., not significant. **(M)** qRT-PCR analysis of *BSP* at indicated samples. **(N)** BSP Immunohistochemical staining and **(O)** Osteocalcin Immunohistochemical staining of hOB TE-bone and hOB+hOC TE-bone untreated and treated with ZA or ALD for 3 days. **(P)** Analysis of osteocalcin gene expression by qRT-PCR and **(Q)** osteocalcin release for both TE-bone models and indicated treatments. Values correspond to the average and standard deviation. Two-tailed Student’s *t*-test was used to determine statistical significance. *p < 0.05; n.s., not significant.

We used decellularized bone matrix as a scaffold and FBS as a medium supplement, by following a previously established a protocol to engineer a human bone construct as a niche for cancer cells (Villasante et al., 2014, 2017). In order to improve the manufacturing process, we implemented a machining process to produce a miniaturized version of our previous bone scaffold to fit in a 96-well plate (Figure 3A).

To evaluate capability of human serum to promote osteoblast and osteoclast differentiation in 3D, we generated two different bone niches using miniaturized bone scaffolds: (i) a monoculture bone with only osteoblasts (hOB), and (ii) a co-culture bone with osteoblasts and osteoclasts (hOB + hOC) (Figure 3B). We evaluated osteoblast and osteoclast differentiation, functionality, and the capability of both engineered tissues to mimic the behavior of native bone. In this regard, we treated the bone niches with two bisphosphonates, Zoledronic acid (ZA) and Alendronate (ALD) that have been shown effective in inhibiting osteoclast function in osteolytic bone diseases in humans and preclinical studies with animals (Farrell et al., 2018).

The hOB+hOC model showed calcium release that was partially inhibited by ZA, but not by ALD (Figure 3C). We found low levels of calcium release and no effect of either drug on the hOB niche (Figure 3C). We also analyzed osteoclast differentiation by TRAP gene expression and TRAP staining (Figures 3D,E). ZA and ALD did not have any effect on TRAP expression and therefore, on osteoclasts maturation (Figure 3D). We performed micro-CT studies to further confirm the maturation of osteoclasts and the effect of bisphosphonates on osteoclasts function in healthy bone. As expected, we observed a trend of decrease in trabecular space and a trend of increase in the trabecular number and connectivity in the hOB+hOC model, compared to the hOC model (Figures 3F,G). We did not find significant effects of ZA or ALD on healthy bone by micro-CT (Figures 3F,G). Together, our results confirmed the capability of hS to substitute FBS in generating healthy, mature and functional osteoclasts within a 3-dimensional bone niche. We also determined the effectiveness of hS for osteoblast differentiation and secretion of the bone organic matrix by osteoblasts within the 3D construct.

Mature osteoblasts secrete large amounts of type I collagen (COL1A1) that is resorbed by osteoclasts. We first tested the capability of the osteoblasts in the hOB and hOB+hOC bone tissues to produce collagenous matrix. Trichrome stains (Figure 3H) and *COL1A1* mRNA expression confirmed that osteoblasts are differentiated and matured, and producing high amounts of type I collagen (Figure 3I). We observed an interesting effect of downregulation of *COL1A1* by ALD on the hOB bone compared to the untreated hOB control (Figure 3I).

We also investigated whether the tissue-engineered bone can mimic the human osteopontin (OPN), bone sialoprotein (BSP) and osteocalcin expression. While osteoblasts express osteopontin, osteoclasts can also express high levels of this matrix protein (Merry et al., 1993). We confirmed that hOB+hOC expressed the *OPN* gene, and that the protein was released at five times higher rates than in the hOB bone (Figures 3J,L). Interestingly, ZA partially inhibited OPN release in the hOB+hOC bone (Figure 3L). On the contrary, ALD induced not only gene expression of *OPN* (Figure 3J), but also production and release of the protein in the hOB bone (Figures 3K,L). Bone sialoprotein (BSP) is mainly secreted by mature osteoblasts, but it was shown that osteoclasts also marginally contribute to the bone matrix composition by expressing this gene (Bianco et al., 1991). As expected, we found a decrease in *BSP* expression in the hOB+hOC bone compared to the hOB bone (Figures 3M,N), which was slightly recovered by ALD treatment (Figure 3M). We finally analyzed the levels of osteocalcin, a matrix protein solely secreted by osteoblasts. Immunohistochemistry and mRNA expression analysis confirmed that osteocalcin was significantly higher expressed in the hOB bone than in the bone niche with osteoclasts (Figures 3O,P). In this case, none of the two drugs affected *BGLAP* expression (Figure 3P) or protein release (Figure 3Q).

### Evaluation of Human Serum as a Substitute of FBS for EWS Cell Culture

**FIGURE 4:**
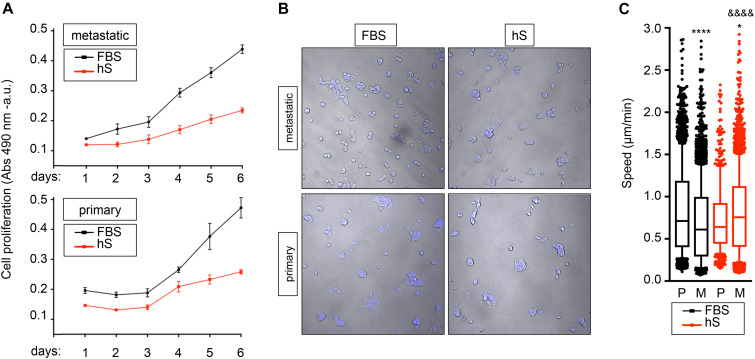
Study of proliferation and migration speed of Ewing’s sarcoma cells cultured in FBS and human serum. **(A)** MTS assay for analyzing cancer cell growth of metastatic (SK-MC) and primary (RD-ES) Ewing’s sarcoma cell lines (*n* = 3–4). Culture medium was supplemented with FBS or human serum (hS). Indicated cells were cultured over 6 days in monolayer. **(B)** representative images of metastatic and primary Ewing’s sarcoma cell lines stained with Hoechst 33342 at indicated culture media in monolayer. **(C)** Box-and-whiskers plot with all of the raw data obtained from LEVER and overlaid to show primary and metastatic cell migration speed under specified conditions. The median is shown by the line bisecting the box. The whiskers represent 10th and 90th percentiles of the data Statistical analysis comparing median speed between conditions is performed using Mann- Whitney test (unpaired, two-tail distribution; confidence level 95%.). Metastatic FBS vs. primary FBS *p* value < 0.0001; *p* value summary ****. Metastatic hS vs. primary hS *p* value = 0.0227; *p* value summary *. Metastatic hS vs. metastatic FBS *p* value < 0.0001; *P* value summary ^&⁣&⁣&&^. Primary hS vs. primary FBS *p* value = 0.0738; *p* value summary = n.s., not significant.

The capacity of metastatic and primary EWS cell lines to proliferate in hS- and FBS-supplemented medium was assessed by MTS assay. As expected, cancer cells survived in both media, but they proliferated slower in hS-medium (Figure 4A). For further characterization of cancer cell behavior, we applied computational image analysis by LEVER to compare cell migration speeds in both media. We labeled metastatic and primary EWS cells with Hoechst 33342 (Figure 4B), and used LEVER for obtaining automate segmentation, tracking, and analysis of migrating cells (Winter et al., 2016). Cell migration speed was higher for metastatic cells than for primary cells cultured in hS-medium (Figure 4C). We obtained the opposite result for the FBS-medium condition (Figure 4C). Together these data suggest that EWS cells better resemble physiological behavior observed in patients in the presence of human serum.

### Generation of Tissue-Engineered Models of Ewing’s Sarcoma

#### Cell Proliferation in 3D Models of Ewing’s Sarcoma

**FIGURE 5:**
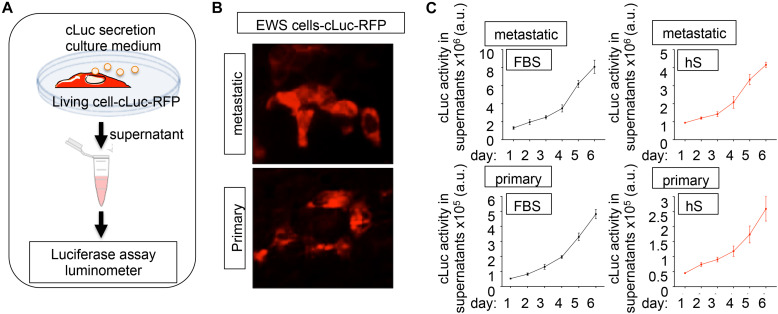
Cypridina Luciferase (cLuc) as a novel strategy for tracking Ewing’s sarcoma cells. **(A)** General scheme of secreted luciferase technology. Cells carrying the cLuc-RFP vector secrete the cypridina luciferase to the culture medium. Luciferase activity can be easily monitored in culture supernatants by using a luminometer. **(B)** Representative fluorescence microscopy images of indicated EWS cells successfully transduced with the cLuc-RFP vector. Positively cLuc-RFP cells are shown in red **(C)** Monitoring of EWS cell growth with the cLuc-RFP reporter. The activity of cLuc was measured in EWS cells cultured during 6 days in FBS and human serum-containing medium by using a luminometer. Values correspond to the average of four independent samples and error bars represent standard deviation.

Cytotoxicity/viability assays and computational image analyses are not easily adapted for 3D cultures, and we lack a robust methodology for analyzing cancer proliferation in 3D studies. Luciferase-based reporters have been widely used to study gene expression at the transcriptional level, to image and monitor tumor growth upon implantation in host animal models, and to evaluate the efficiency of anticancer drugs in monolayer cell cultures (Ow et al., 1986; Badr, 2014; Ohmiya, 2015; Kaskova et al., 2016; Matta et al., 2018). We implemented luminescence as a tool for non-destructive tracking cancer cells over time in the bone-engineered niche. We generated a primary and a metastatic EWS cell line expressing a novel secreted luciferase, known as Cypridina Luciferase (cLuc) (Figures 5A,B). cLuc has the advantage that its activity can be easily quantified in cell culture supernatants, and that signaling is directly proportional to the number of EWS cells in culture (Figure 5B). To monitor cancer cell proliferation, we cultured both EWS cell lines in 2D for 6 days and confirmed that EWS cells grow slower in hS-medium (Figure 5C). We concluded that cLuc is a robust tool that can be used for tracking cells and their responses to drugs in a non-destructive fashion.

#### Metastatic Model of Ewing’s Sarcoma Cultured in Human Serum

**FIGURE 6:**
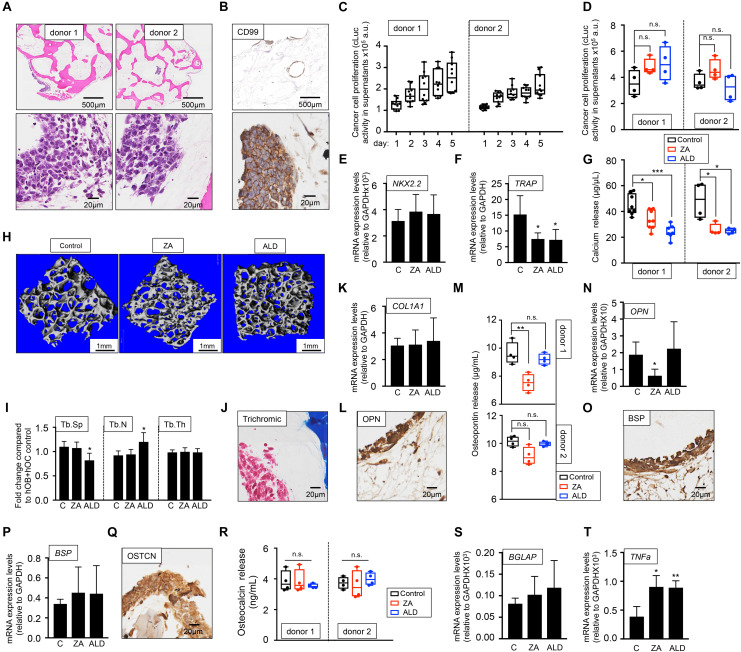
Evaluation of the effect of bisphosphonates on the tissue-engineered metastatic model of Ewing’s sarcoma cultured in human serum-containing medium. **(A)** Hematoxylin and eosin staining of the tissue-engineered metastatic model of Ewing’s sarcoma (TE-metastatic). One donor of hMSC for osteoblasts differentiation and two donors of monocytes CD14^+^ (osteoclasts precursors) were assessed to build two TE-metastatic models (donor 1 and donor 2). Human serum was used as a supplement to culture medium in all cases. **(B)** Representative image of immunohistochemical staining of the TE-metastatic (donor 1) model for Ewing’s sarcoma marker CD99. Counterstaining was performed with hematoxylin QS (blue). **(C)** Analyses of EWS cell proliferation within the bone niche over 5 days by quantifying cLuc activity in the indicated TE-metastatic models. **(D)** Effect of zoledronic acid (ZA) and Alendronate (ALD) on EWS cell proliferation within the 3-dimensional bone niche. Metastatic EWS aggregates were cultured within the TE-bone containing osteoblasts and osteoclasts for 5 days and then, they were treated with 20 μM ZA or 10 μM ALD for 3 days. Cancer cell proliferation was determined by cLuc activity in supernatants using a luminometer. *n* = 4 per condition were tested. Values correspond to the average of cLuc activity in each condition and error bars represent standard deviation. **(E)** mRNA levels determined by qRT-PCR of the Ewing’s sarcoma markers NKX2.2, **(F)** and osteoclast marker TRAP from the indicated groups [C, control without treatment (donor1, *n* = 3); ZA, treated with zoledronic acid (donor1, *n* = 4); ALD, treated with alendronate (donor1, *n* = 4)]. mRNA levels were normalized to GAPDH levels. Error bars represent standard deviation of relative expression *n* = 4. **(G)** Calcium release levels from the indicated conditions (*n* = 7–8 donor 1; *n* = 3–4 donor 2). Two-tailed Student’s *t*-test was used to determine statistical significance. *p < 0.05; ***p < 0.001. **(H)** Representative micro CT images from Donor 1 TE-bone constructs with or without bisphosphonates treatment (*n* = 4 per condition). **(I)** Plots of the structural parameters trabecular space (Tb.Sp), trabecular number (Tb.N) and connectivity density (Conn.Dens) from microCT images for the groups examined (*n* = 4 per group). **(J)** Representative Masson’s trichrome staining images showing collagen in blue the TE-metastatic control model. **(K)** qRT-PCR analysis of *COL1A1*. Fold change was calculated by normalizing to GAPDH levels in individual samples. Values correspond to the average and standard deviation. C, control *n* = 3; ZA, Zoledronic acid treatment *n* = 4; ALD, Alendronate treatment *n* = 4. **(L)** Representative image of OPN Immunohistochemical staining of the TE-metastatic control model. Counterstaining was performed with hematoxylin QS (blue). **(M)** Osteopontin release levels from the metastatic model at indicated conditions. Error bars represent standard deviation; *n* = 4 per condition. Two-tailed Student’s *t*-test was used to determine statistical significance. **p < 0.01; n.s., not significant. **(N)** Expression levels of *OPN* by qRT-PCR in the donor 1 model and indicated treatments. mRNA levels were normalized to GAPDH levels. Error bars represent standard deviation of relative expression. Two-tailed Student’s *t*-test was used to determine statistical significance; *p < 0.05. **(O)** Representative image of BSP Immunohistochemical staining of the TE-metastatic control model. **(P)** qRT-PCR analysis of *BSP* at indicated samples. C, control *n* = 3; ZA, Zoledronic acid treatment *n* = 4; ALD, Alendronate treatment *n* = 4. **(Q)** Osteocalcin Immunohistochemical staining of control **(R)** Analysis of osteocalcin release, and **(S)** osteocalcin gene expression by qRT-PCR for the indicated models and treatments. Values correspond to the average and standard deviation. Two-tailed Student’s *t*-test was used to determine statistical significance. n.s., not significant. **(T)** Expression levels of *TNFα* by qRT-PCR in the donor 1 model and indicated treatments. mRNA levels were normalized to GAPDH levels. Error bars represent standard deviation of relative expression. Two-tailed Student’s *t*-test was used to determine statistical significance. *p < 0.05; **p < 0.01.

To investigate whether EWS can proliferate in the bone niche cultured with human serum and activate the vicious cycle observed in patients, we first introduced the metastatic EWS cell line expressing cLuc in the hOB-hOC bone. Two different bone niches containing osteoclasts differentiated from two monocytes donors were generated (donor 1 and donor 2). EWS cells formed 3D aggregates within the bone niche (Figure 6A) that expressed the EWS marker CD99 (Figure 6B). We also confirmed that the tumors were proliferating by quantifying cLuc activity in supernatants over-time (Figure 6C), without significant differences in proliferation rates between metastatic EWS cells within the bone niches of donor 1 and donor 2 (Figure 6C). Treatment with bisphosphonates, ZA and ALD, did not affect metastatic EWS cell survival, as demonstrated by cLuc activity (Figure 6D). mRNA expression of the EWS marker NKX2.2 neither was affected by drug treatment (Figure 6E). However, ZA and ALD had a significant effect on osteoclast biology and bone resorption when metastatic EWS cells are in the bone niche. Both drugs inhibited expression of the TRAP gene (Figure 6F), and impaired calcium release in bone niches (Figure 6G). Micro-CT analyses demonstrated that ALD blocked osteoclast activity, as evidenced by decreased trabecular spaces and trabecular numbers in the bone constructs, compared to the untreated control (Figures 6H,I). However, ZA did not show any effect on the metastatic bone niches (Figures 6H,I).

As for the healthy bone niche (Figure 3), we also investigated the effect of ZA and ALD on osteoblasts biology and bone matrix production in the presence of metastatic EWS cells. We first confirmed type I collagen expression by Masson trichrome staining (Figure 6J). Next, we treated the model with ZA and ALD, and found that *COL1A1* expression levels were unaffected by the treatment with bisphosphonates (Figure 6K).

Osteopontin (OPN) was expressed both in osteoblasts and osteoclasts, and in the metastatic bone model, as evidenced by immunohistochemistry (Figure 6L), release studies (Figure 6M), and by qRT-PCR (Figure 6N). Treatment with ZA effectively reduced osteopontin release (Figure 6M) and gene expression (Figure 6N) in one of the two donors assessed. Expression of bone sialoprotein (Figures 6O,P) and osteocalcin (Figures 6Q–S) were not altered by bisphosphonates, compared to the untreated control.

TNFα is a cytokine produced by macrophages, monocytes and T cells. A number of studies demonstrated that bisphosphonates trigger TNFα expression (Sauty et al., 1996; Dicuonzo et al., 2003) by promoting the conversion of osteoclast progenitors into TNFα macrophages expressing cells, which impairs osteoclasts differentiation and maturation (Morita et al., 2017). TNFα is also expressed by Ewing’s sarcoma primary tumors at higher levels than the metastatic disease (Rube et al., 2003). However, the activity of TNFα in Ewing’s sarcoma is controversial (Patio-Garcia et al., 2000). We found that TNFα expression in tumor models was upregulated by bisphosphonates, as described for other physiological systems (Figure 6T).

#### Primary Model of Ewing’s Sarcoma Cultured in Human Serum

**FIGURE 7:**
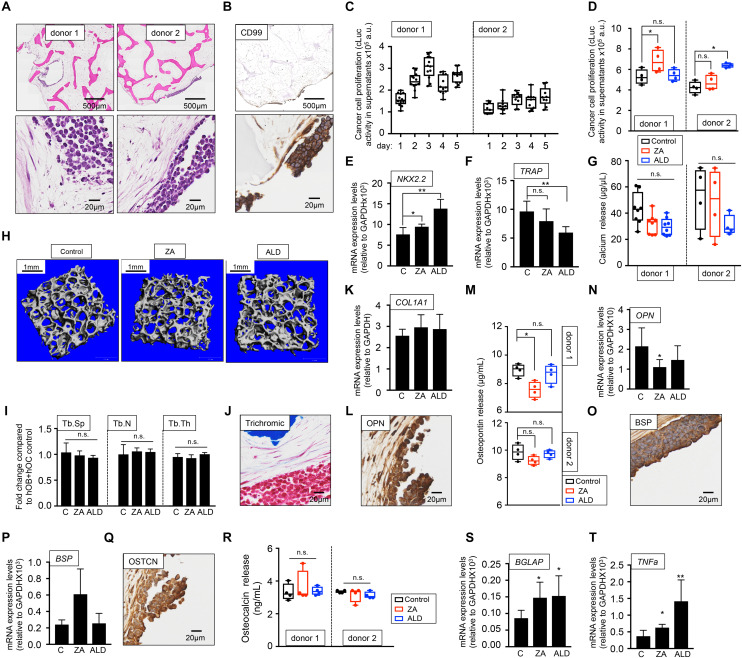
Evaluation of the effect of bisphosphonates on the tissue-engineered primary model of Ewing’s sarcoma cultured in human serum-containing medium. **(A)** Hematoxylin and eosin staining of the tissue-engineered metastatic model of Ewing’s sarcoma (TE-primary). One donor of hMSC for osteoblasts differentiation and two donors of monocytes CD14^+^ (osteoclasts precursors) were assessed to build two TE-primary models (donor 1 and donor 2). Human serum was used as a supplement to culture medium in all cases. **(B)** Representative image of immunohistochemical staining of the TE-metastatic (donor 1) model for Ewing’s sarcoma marker CD99. Counterstaining was performed with hematoxylin QS (blue). **(C)** Analyses of EWS cell proliferation within the bone niche over 5 days by quantifying cLuc activity in the indicated TE-primary models. **(D)** Effect of zoledronic acid (ZA) and Alendronate (ALD) on EWS cell proliferation within the 3-dimensional bone niche. Primary EWS aggregates were cultured within the TE-bone containing osteoblasts and osteoclasts for 5 days and then, they were treated with 20 μM ZA or 10 μM ALD for 3 days. Cancer cell proliferation was determined by cLuc activity in supernatants using a luminometer. *n* = 4 per condition were tested. Values correspond to the average of cLuc activity in each condition and error bars represent standard deviation. **(E)** mRNA levels determined by qRT-PCR of the Ewing’s sarcoma markers NKX2.2, **(F)** and osteoclast marker TRAP from the indicated groups [C, control without treatment (donor1, *n* = 4); ZA, treated with zoledronic acid (donor1, *n* = 4); ALD, treated with alendronate (donor1, *n* = 4)]. mRNA levels were normalized to GAPDH levels. Error bars represent standard deviation of relative expression. Two-tailed Student’s *t*-test was used to determine statistical significance *p < 0.05; **p < 0.01; n.s., not significant. **(G)** Calcium release levels from the indicated conditions (*n* = 8 donor 1; *n* = 4 donor 2). Two-tailed Student’s *t*-test was used to determine statistical significance, n.s., not significant. **(H)** Representative micro CT images from Donor 1 constructs with or without bisphosphonates treatment (*n* = 4 per condition). **(I)** Plots of the structural parameters trabecular space (Tb.Sp), trabecular number (Tb.N) and connectivity density (Conn.Dens) from microCT images for the groups examined (*n* = 4 per group). Two-tailed Student’s *t*-test was used to determine statistical significance, n.s., not significant. **(J)** Representative Masson’s trichrome staining images showing collagen in blue the TE-primary control model. **(K)** qRT-PCR analysis of *COL1A1*. Fold change was calculated by normalizing to GAPDH levels in individual samples. Values correspond to the average and standard deviation. C, control *n* = 4; ZA, Zoledronic acid treatment *n* = 4; ALD, Alendronate treatment *n* = 4. **(L)** Representative image of OPN Immunohistochemical staining of the TE-primary control model (Donor 1). Counterstaining was performed with hematoxylin QS (blue). **(M)** Osteopontin release levels from the primary models at indicated conditions. Error bars represent standard deviation; *n* = 4 per condition. Two-tailed Student’s *t*-test was used to determine statistical significance. *p < 0.05; n.s., not significant. **(N)** Expression levels of *OPN* by qRT-PCR in the donor 1 model and indicated treatments. mRNA levels were normalized to GAPDH levels. Error bars represent standard deviation of relative expression. Two-tailed Student’s *t*-test was used to determine statistical significance; *p < 0.05. **(O)** Representative image of BSP Immunohistochemical staining of the TE-primary control model. **(P)** qRT-PCR analysis of *BSP* at indicated samples. C, control *n* = 4; ZA, Zoledronic acid treatment *n* = 4; ALD, Alendronate treatment *n* = 4. **(Q)** Osteocalcin immunohistochemical staining of control model. **(R)** Analysis of osteocalcin release, and **(S)** osteocalcin gene expression by qRT-PCR for the indicated models and treatments. Values correspond to the average and standard deviation. Two-tailed Student’s *t*-test was used to determine statistical significance. *p < 0.05; n.s., not significant. **(T)** Expression levels of *TNFα* by qRT-PCR in the donor 1 model and indicated treatments. mRNA levels were normalized to GAPDH levels. Error bars represent standard deviation of relative expression. Two-tailed Student’s *t*-test was used to determine statistical significance. *p < 0.05; **p < 0.01.

As for the metastatic model, two sources of osteoclasts-differentiated monocytes were tested, and two primary tumor models were built. We confirmed the presence of tumor aggregates within the bone niche in both models (Figure 7A) and expression of the CD99 EWS marker (Figure 7B) and found disparities in EWS cell proliferation between bone niches (Figure 7C). Unexpectedly, treatment with bisphosphonates increased cLuc activity (Figure 7D) and expression of EWS marker NKX2.2 (Figure 7E), suggesting that ZA and ALD favored tumor proliferation. Although ALD reduced TRAP gene expression (Figure 7F), none of the drugs had an effect on bone resorption, as demonstrated by calcium release assays (Figure 7G) and microCT analyses (Figures 7H,I).

These results show that not only bisphosphonates are not effective in blocking bone resorption in the EWS primary tumor model, but also enhance cancer cell proliferation. We also investigated the possible effect of ZA and ALD on osteoblasts and bone matrix. Bisphosphonates treatment did not affect total collagen levels compared to the untreated control (Figures 7J,K). ZA treatment altered osteopontin in donor 1 only (Figures 7L–N). No effect was observed for BSP (Figures 7O,P), and they neither had an effect on osteocalcin protein (Figures 7Q,R), but they both upregulated osteocalcin mRNA expression levels (Figure 7S). Importantly, total TNFα expression levels were upregulated in the primary EWS models treated with both bisphosphonates, as observed in the metastatic models (Figure 7T).

## Discussion

We previously generated human tissue-engineered models of Ewing’s sarcoma within its bone niche (Villasante et al., 2014, 2017), using an approach that involved the 2D culture and *in vitro* expansion of hMSC, human monocytes and human cancer cell lines, for differentiation within 3D bone scaffold (Villasante et al., 2017). For the *ex-vivo* culture of cells we used basal culture media with supplements, FBS being the most important one. In an effort to improve our tumor-engineered models in terms of biomimicry, we now analyze whether human derivatives better emulate human *in situ* environments than FBS, in a robust and reproducible manner.

We initially studied the capability of human serum and human platelet lysate to support osteoblasts and osteoclasts differentiation. As hypothesized, the differential effects of FBS, hS or hP media on cells were evident. During osteoblasts differentiation, FBS-OB and hS-OB media acted in a very similar manner, while hP-OB medium was not effective. Interestingly, control medium containing hS induced alkaline phosphatase (ALP) expression without inducing a complete hMSC differentiation into mature osteoblasts. Human serum-mediated expression of ALP was also described for human adipose stem cells cultures, but the molecular mechanism driving this phenomenon has not been thoroughly investigated (Kyllonen et al., 2013).

One of the major challenges for the establishment of robust and successful protocols for cell differentiation is to obtain the cell types required with high efficiency. The gold standard method for generating osteoclasts *in vitro* consists in differentiating osteoclasts from human monocytes using RANKL and M-CSF in FBS-based medium. We and others demonstrated high yields of mature osteoclasts by following this procedure. In the present study, we report that even higher osteoclast differentiation efficiency was observed in the differentiation medium containing human serum. In fact, CD14 mRNA levels were lower in cells cultured with control medium with only hS than with differentiation medium containing FBS.

Of note, we found that the remaining undifferentiated cells acquired an M1 macrophage phenotype [physiological osteoclasts precursors (Huang et al., 2017)] when cultured in hS-OC medium, while FBS-OC induced M2 phenotype. Further studies will be necessary to elucidate the role of M1 and M2 phenotypes in osteoclasts differentiation. However, based on our data, it is possible that human serum could prime monocytes to M1 phenotype, which will become mature osteoclasts by the presence of RANKL in the differentiation medium. As shown, RANKL is also effective in FBS-OC medium. However, FBS favors M2 phenotype. M2 cells have not been reported as osteoclast precursors capable to generate mature osteoclast (Huang et al., 2017). Therefore, the presence of M2 cells in the culture could decrease osteoclast differentiation efficiency, which is in fact what we experimentally observed.

Based on data from Figures 1A,B, we can also argue that hP-OC medium could favor M1 phenotype due to (i) most of the cells that do not resemble osteoclasts are round cells (similar to those observed for human serum treatment), and (ii) cells observed in Figure 1A are not CD14+ monocytes because this marker is expressed at low levels with the differentiation treatment supplemented with PL (Figure 1B).

After choosing human serum as the most suitable candidate for supplementing the culture medium, we built a 3D tissue-engineered bone. We confirmed that the engineered bone is a living tissue and mimics human native bone in the regard of cell composition and functionality and mineralized and non-mineralized matrix composition. For further validation of the tissue-engineered bone, we evaluated healthy bone cell responses to two bisphosphonates (BPs), Zoledronic acid (ZA) and Alendronate (ALD), which have been shown effective in inhibiting osteolysis in humans and/or preclinical studies of disease. BPs are generally safe, but hypocalcemia may result from the treatment with bisphosphonates, particularly with ZA, which exhibits the most potent pharmacological action and affinity to bones. In line with the physiological adverse reaction of BP, we found that ZA had an important effect on reducing calcium release in the engineered-bone containing osteoclasts and osteoblasts, while ALD did not induce any adverse effect. Osteopontin is one of the key components in osteoclast attachment to bone during bone resorption. As expected, osteopontin release was also blocked by the most potent BP assessed, which further confirmed the biomimicry of the *in vitro* bone generated in the context of human serum. Interestingly, we found that ALD induced the increase of osteopontin expression and release by osteoblasts, which in turn enhanced matrix formation. This result suggests a novel role for ALD on directly affecting osteoblasts biology to produce bone matrix.

Traditionally, evaluation of anticancer efficacy of selected drugs has been done in 2D-cultured cells using cell-based assays to determine the pharmacological effect on cell growth and survival. For example, MTT, MTS or Alamar Blue are the most common cell-based assays to analyze cell viability and to measure the toxicity to cells of anticancer drugs *in vitro* (Riss et al., 2004). Unfortunately, these routinely used cytotoxicity/viability assays for 2D cultures failed in 3D systems, and are not easily adapted for 3D cultures studies (Friedrich et al., 2007; Celli et al., 2014; Zanoni et al., 2016). The lack of methodology for drug testing in 3D systems has stimulated the development of new assays specifically designed for 3D culture models, and the adaptation of biological tools that were not intended to be used for determining drug efficacy in 3D. This last one is the case of bioluminescent techniques based on the production of light by luciferase-catalyzed reactions (Kaskova et al., 2016). Recently, a new generation of luciferases characterized by being secreted to the culture medium has become available (Falcone and Casucci, 2016; Kaskova et al., 2016). In this study, we established a novel luciferase-based system to track cell growth and evaluate the effect of drugs on EWS cells, in co-culture with osteoblasts and osteoclasts, in a 3D bone-engineered microenvironment. We introduced a secreted luciferase (cLuc) in EWS cells for monitoring cancer cell proliferation over-time and in a non-destructive fashion by quantifying luciferase activity in cell culture supernatants. We confirmed for the first time the use of cLuc as a robust tool for evaluation of therapeutic compounds in *in vitro* tissue-engineered systems.

By using the cLuc tool, we addressed the effect of bisphosphonates on cancer cell proliferation. The current protocol for Ewing sarcoma patients is Euro EWING 2012. The ZA randomization (R2) branch of the protocol is defined to determine whether the addition of this bisphosphonate to consolidation chemotherapy is associated with improved clinical outcome in patients. Thus, we studied the effects of ZA on the tissue-engineered models of EWS, and also ALD effects as an alternative BP for comparison. We found disparities in cell behavior and drug responses between metastatic and primary EWS when introduced in the bone-engineered niche. In the metastatic model, both bisphosphonates blocked calcium release in the two osteoclasts donors, and they did not affect cancer cell proliferation. However, none of the treatments was effective in inhibiting bone resorption in the primary tumor model. Moreover, they triggered cancer cell proliferation (ZA in donor 1, ALD in donor 2).

Few preclinical models of EWS have been developed, limiting the number of studies of effectiveness of bisphosphonates and their basic mechanisms of action in EWS. Among these studies, ZA has demonstrated to be a potentially interesting tool for therapeutic application in models of EWS cell lines in monolayer and metastatic mouse models. Zhou et al. (2005) showed that ZA inhibits tumor growth in a mouse model generated by intratibia injection of bone metastatic TC71 cells. Odri et al. analyzed the potential therapeutic efficacy of ZA alone or in combination with ifosfamide (Odri et al., 2010) and observed that EWS cell lines have different sensitivities to ZA. For example, A-673 was sensitive to ZA (IC50 = 3 μM) and TC-71 was more resistant (IC50 = 100 μM). They also demonstrated that ZA alone prevents EWS development in a mouse model of A673 cells injected in the medullar cavity of the tibia of immunodeficient mice (Odri et al., 2010).

Using the same approach and cell lines, A673 and TC-71, they found that ZA inhibits pulmonary metastasis dissemination (Odri et al., 2014). A673 is an EWS cell line which primary site of origin is the muscle, while TC-71 is an originally metastatic tumor located in the humerus (May et al., 2013). In one study, ZA did not inhibit tumor progression when A673 cells were injected in the mouse soft tissue (Odri et al., 2010). This is maybe due to A673 were growing in their native-like niche (soft tissue), as we observed for RD-ES cells in our primary tumor model. However, ZA had an important effect when the bone metastatic cell line TC-71 was injected in the bone mouse, as well as when the muscle A673 cell line is artificially introduced (“metastasizes”) in the tibia. Together, our data and previously published reports suggest that bisphosphonates, and ZA in particular, have a therapeutic effect in metastatic models of EWS. In this study, we developed the first primary EWS tumor model within its human bone niche and cultured with human serum. By using this model, our data suggest that treatment with bisphosphonates could not be effective in inhibiting bone resorption in primary EWS. However, much research will be necessary to unravel the bisphosphonate efficacy or non-efficacy in primary EWS. In addition, the results of this study suggest advantages of using human serum in studies of other tumors and pharmacological systems.

## Data Availability Statement

The raw data supporting the conclusions of this article will be made available by the authors, without undue reservation.

## Author Contributions

AV conceived the project, performed the experiments, collected and analyzed the data, performed the analysis, and wrote the manuscript. SR, AC, and RL performed experiments and/or collected the data. GV-N revised the manuscript. All authors contributed to the article and approved the submitted version.

## Conflict of Interest

The authors declare that the research was conducted in the absence of any commercial or financial relationships that could be construed as a potential conflict of interest.

## Footnotes

1 http://pga.mgh.harvard.edu/primerbank/
